# Visualizing Metal Content and Intracellular Distribution in Primary Hippocampal Neurons with Synchrotron X-Ray Fluorescence

**DOI:** 10.1371/journal.pone.0159582

**Published:** 2016-07-19

**Authors:** Robert A. Colvin, Qiaoling Jin, Barry Lai, Lech Kiedrowski

**Affiliations:** 1 Department of Biological Sciences, Interdisciplinary Graduate Program in Molecular and Cellular Biology, Neuroscience Program, Ohio University, Athens, Ohio, United States of America; 2 Department of Physics & Astronomy, Northwestern University, Evanston, Illinois, United States of America; 3 X-ray Science Division, Advanced Photon Source, Argonne National Laboratory, Lemont, Illinois, United States of America; 4 The Psychiatric Institute, Departments of Psychiatry and Pharmacology, the University of Illinois at Chicago, Chicago, Illinois, United States of America; CINVESTAV-IPN, MEXICO

## Abstract

Increasing evidence suggests that metal dyshomeostasis plays an important role in human neurodegenerative diseases. Although distinctive metal distributions are described for mature hippocampus and cortex, much less is known about metal levels and intracellular distribution in individual hippocampal neuronal somata. To solve this problem, we conducted quantitative metal analyses utilizing synchrotron radiation X-Ray fluorescence on frozen hydrated primary cultured neurons derived from rat embryonic cortex (CTX) and two regions of the hippocampus: dentate gyrus (DG) and CA1. Comparing average metal contents showed that the most abundant metals were calcium, iron, and zinc, whereas metals such as copper and manganese were less than 10% of zinc. Average metal contents were generally similar when compared across neurons cultured from CTX, DG, and CA1, except for manganese that was larger in CA1. However, each metal showed a characteristic spatial distribution in individual neuronal somata. Zinc was uniformly distributed throughout the cytosol, with no evidence for the existence of previously identified zinc-enriched organelles, zincosomes. Calcium showed a peri-nuclear distribution consistent with accumulation in endoplasmic reticulum and/or mitochondria. Iron showed 2–3 distinct highly concentrated puncta only in peri-nuclear locations. Notwithstanding the small sample size, these analyses demonstrate that primary cultured neurons show characteristic metal signatures. The iron puncta probably represent iron-accumulating organelles, siderosomes. Thus, the metal distributions observed in mature brain structures are likely the result of both intrinsic neuronal factors that control cellular metal content and extrinsic factors related to the synaptic organization, function, and contacts formed and maintained in each region.

## Introduction

Metallomics is a rapidly evolving and growing science fueled by a new awareness of the roles played by various metals in the physiology of living tissues, microorganisms, and in human disease [1, 2]. The development of quantitative tools such as synchrotron radiation X-ray fluorescence (SRXRF), laser ablation inductively coupled plasma mass spectrometry (LA-ICP-MS), and proton induced X-ray emission (PIXE) are allowing accurate and precise measurements of the total metal contents and topography of tissues, single cells and organelles [3–6]. The hippocampal formation has a striking and much studied metal distribution [7–10] that reflects its highly ordered and laminated structure and synaptic organization [11]. Zinc is concentrated in the hippocampal mossy fiber pathway and begins to accumulate there during early development [12]. The mossy fiber pathway originates in the dentate gyrus and projects into the hilus and stratum lucidum of CA3 and gives the mature rodent hippocampus its characteristic and well documented zinc profile with Timm’s stain [7]. The mossy fiber boutons contain glutamatergic and zinc containing vesicles. This zinc results in higher levels of zinc in the dentate gyrus and CA3, generally higher than any other region of the brain. This zinc is responsible, at least in part, for the selective vulnerability of the CA3 region to ischemia, stress, epilepsy, aging, and neurodegenerative and neuropsychiatric disorders [13, 14]. Manganese and iron show spatial correlations with zinc as determined by SRXRF of hippocampal slices [10, 15, 16]. While the characteristic high zinc levels of the CA3 are primarily the result of zinc accumulated in the boutons [7], it is not thought that manganese or iron accumulates in synaptic vesicles. It is generally believed, but not proven, that neurons of the CA1 and CA3 contain similar total levels of zinc and other metals. Thus, we sought to determine if the metal content and subcellular distribution observable in individual cultured primary neurons derived embryonically from these regions would be consistent with this belief. In the present study, we determined the average metal content of individual soma and subcellular distribution of several metals using cultured primary neurons derived from the hippocampal formation and compared those with neurons derived from the cortex, which have been much more extensively characterized [17, 18].

## Methods

### Primary Neuron Cultures

The cultures were prepared using custom-ordered cryopreserved SPOT^™^ kits distributed by the University of Illinois Research Resources Center (http://www.rrc.uic.edu/portal/SPOT_Culture_Kit). Kit production was approved by the Office of Animal Care and Institutional Biosafety at the University of Illinois at Chicago. The cortical and hippocampal tissue used for production of these kits was dissected from the brains of E19 Sprague-Dawley rats. To separate the CA1 region from the dentate gyrus (DG), the hippocampi were unfolded and divided longitudinally along the border between the CA2 and CA3 regions. The cells from SPOT^™^ kits were seeded on silicon nitride windows (SiN, Silson, Ltd. UK) that were coated with a polyethyleneimine [17] solution to improve cell adherence. To culture the neurons, neurobasal medium supplemented with 2% B27 and 2 mM glutamine was used. The cells were cultured in a humidified incubator maintained at 37°C and 5% O_2_, 5% CO_2_ and 90% N_2_ atmosphere [19, 20].

### Freezing and Cryogenic Storage of Hydrated Neurons

After 4–6 days *in vitro* when process growth had mostly stabilized, experimental manipulations were performed. The SiN windows with neurons attached were carefully lifted from the culture dish and rinsed two times by gently dipping into an Eppendorf tube containing 1 ml of freshly prepared ice-cold wash buffer (154 mM NaCl, 5.6 mM KCl, 26 mM NaHCO_3_, 100 μM EDTA, chelex treated, pH 7.4) [21]. Excess liquid on the backside of the window (non-cell side) was removed by blotting with filter paper. The windows were then attached to the tweezers supplied with a FEI Vitrobot Mark IV plunge freezer. Plunge freezing was carried out by plunging cells into liquid-nitrogen cooled liquid ethane following the manufacturer’s instructions with chamber set at 30°C, 100% humidity, and with the following blotting parameters: blot time = 2 s, blot force = 0 mm, and blot total = 1.

Subsequently, SiN windows were transferred from liquid ethane to liquid nitrogen and detached from the tweezers. The frozen hydrated cells on SiN windows were imaged using a 20x objective in an integrated cryo light microscope (Nikon 50i light fluorescent microscope equipped with an Instec CLM77K cryostage) around -160°C. Coordinates for neurons of interest were recorded relative to corners of the SiN window. After observation, SiN windows were vertically inserted into a custom window holder with defined orientation and stored in liquid nitrogen until they were retrieved for SRXRF imaging.

### Synchrotron Radiation X-Ray Fluorescence (SRXRF) Analysis of Frozen Hydrated Single Neurons

The equipment required for SRXRF analysis is available as part of the general user facility at the Advanced Photon Source (APS, Argonne National Laboratory, Argonne, IL) beamline 2-ID-D. Upon mounting onto a custom holder in a liquid nitrogen bath, the neuron-containing SiN window was quickly placed in the center of a Cryojet (Oxford Instruments) that convectively cooled the sample with cold nitrogen gas maintained at 100°K. To prevent frosting of the sample, the cold nitrogen jet was surrounded by a dry nitrogen jet at ambient temperature placed inside a chamber with a continuous flow of dry nitrogen. A monochromatic X-ray beam was focused on the specimen using a Fresnel zone plate. Energy of the incident X-ray beam was 10 keV allowing excitation of zinc, potassium, sulfur, and other transition elements. A scan area containing neurons of interest was located by the coordinates obtained from the observation in cryo light microscope and further defined by a coarse 2-D scan viewed using the MAPS software (see below). A final high resolution scan was performed with a pixel step size of 0.25 μm and a 250 msec dwell time.

### SRXRF Data Analysis

Because the X-ray fluorescence energy is specific for each element, the spectrum provides unambiguous information about elemental contents of the scanned neuron. Spectral fitting and background correction of the elemental fluorescence peaks were performed as described previously [22]. Elemental fluorescence peak signals were calibrated with a NIST (National Institute of Standards and Technology, Gaithersburg, MD) standard prior to a scan session. The detector has very low noise which enables it to count individual XRF photons. The main contribution of analytical variance is photon noise from Poisson statistic, which is sqrt(XRF_count) and is generally 5% or less.

Analysis of the raw data 2-D image was performed with the MAPS software [23]. The MAPS software has a drawing tool that allows an ROI to be manually drawn around any region in the scan and quantified. In addition the MAPS software allows line scan data to be obtained from 2-D scans by manually defining the limits of a single line drawn on the scan image. The MAPS software reports fluorescence intensities for each element along the trajectory of that line.

A variable amount of extracellular metals unavoidably remains after the washing procedures, this sample background was estimated for each SiN window using the following procedure. The high resolution 2-D scan for potassium was used to identify a region without cellular material. This region was carefully outlined manually as a region of interest (ROI) and the average metal content calculated (μg/cm^2^) for each metal analyzed. This value was subtracted from the average metal content obtained from ROIs drawn around the soma or subcellular structures of the same scan. This method is utilized since the sample background ROI was subjected to all the same experimental treatments as regions composed of cellular material.

### Statistical Analysis

As SRXRF analysis of freeze-dried primary cortical neurons has been replicated many times by the authors [17], considerations of efficient allocation of resources and beamtime guided the decision not to generate biological replicates of the studies reported here. Still, the authors are confident that a random sample of cells from a single culture (as done in these studies) represents the characteristics of the population as a whole. Based on our experience, biological replicates generally have similar characteristics. It is reasonable to assume that the characteristics of individual neurons on a coverslip will vary independently and variability will necessarily be due to various factors of the culture conditions (both extrinsic and intrinsic to the neurons) acting independently—this strongly predicts a Gaussian distribution of measured parameters. However, because of the small N of our data, we cannot know with certainty that our data set is Gaussian. Thus, we used a nonparametric test—Kruskal-Wallis test with Dunn's Multiple Comparison Test to test significance (GraphPad Prism version 4.03 for Windows, GraphPad Software, San Diego California USA). In figures where the mean average metal contents of ROIs are compared, these data were derived from manually drawn ROIs. The number of individual neurons or ROIs used to calculate the mean is indicated in the figure legend.

## Results

### Subcellular Distributions of Metals Observed in the Soma of Cultured Primary Neurons Derived from the Hippocampus

**Fig 1:**
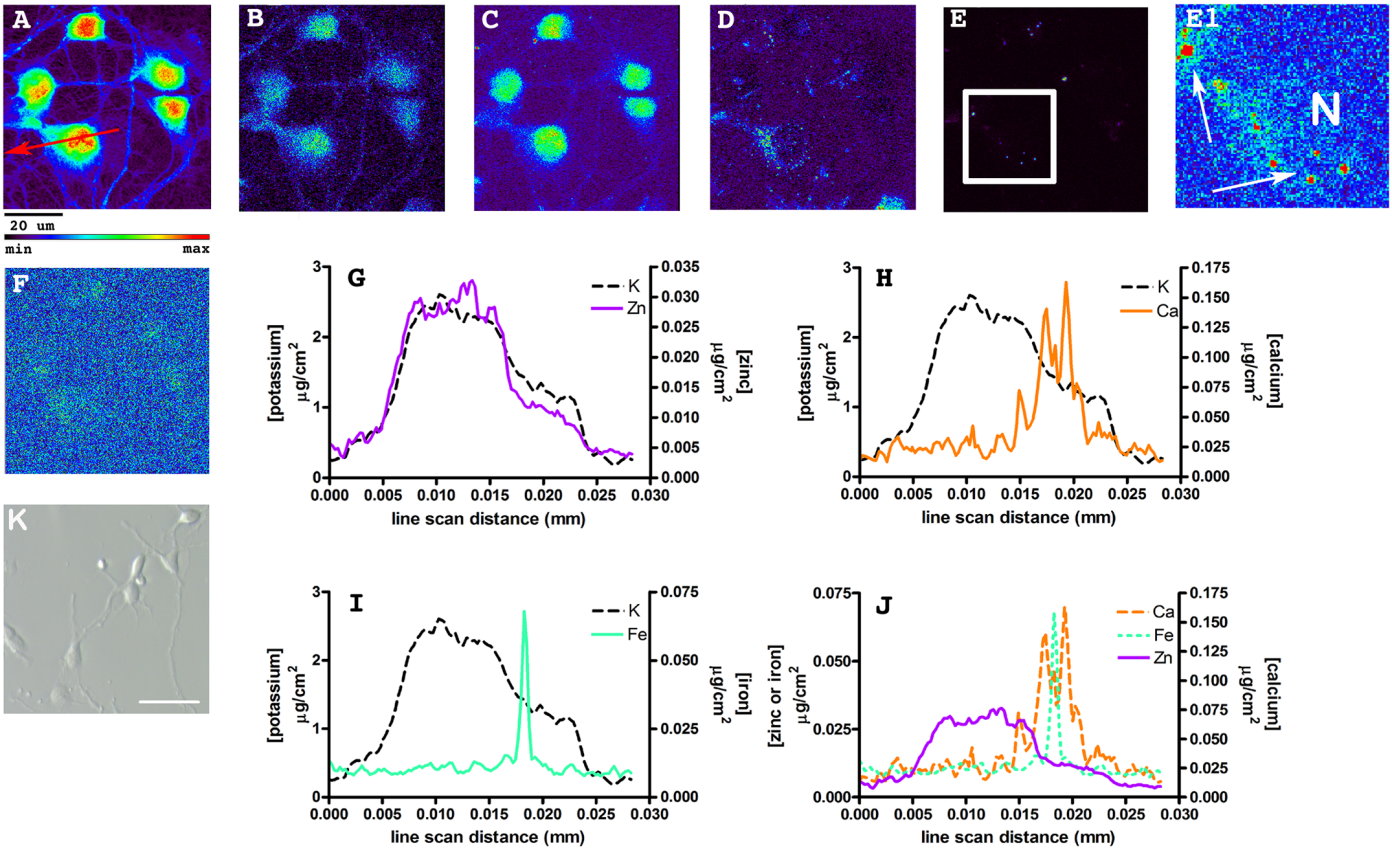
2-D scans and line scan data derived from SRXRF analysis of frozen hydrated primary neurons cultured from the dentate gyrus. The relative intensity rainbow scale used is shown below the potassium scan (A)–red is highest, black is lowest– μg/cm^2^. Image size calibration is shown as a black bar, below the potassium scan. (A) Potassium, (B) sulfur, (C) zinc, (D) calcium, (E) iron, and (F) manganese. (E1) Shows a blow-up of the region outlined in the white box in E illustrating iron puncta and N represents the approximate location of the nucleus. In E1 the scale is changed to allow better visualization of the iron distribution in the soma. Using this scale, the iron puncta are over-saturated (see white arrows pointing to red puncta). Shown in the potassium 2-D scan (A) is a red arrow that illustrates the location and direction of the line scan that produced the plots: (G) zinc and potassium; (H) calcium and potassium; (I) iron and potassium; (J) iron, calcium and zinc. The maximum analytical variance calculated for these data: K = 0.13 μg/cm^2^, Ca = 0.03 μg/cm^2^, Fe = 0.01 μg/cm^2^, Zn = 0.005 μg/cm^2^. Note that the 2-D scans and line scan data are not sample background corrected. Sample backgrounds for this SiN window were calculated as (μg/cm^2^): potassium = 0.194; sulfur = 0.034; zinc = 0.005; calcium = 0.02; iron = 0.009; and manganese = 0.004. (K) Light microscopic image of dentate gyrus neurons prior to flash freezing. Images were taken using 20x NA 0.3 Zeiss LD A-Plan Hoffman Modulation Contrast lens and Zeiss Axiovert S100 microscope controlled by Zeiss Axiovision v. 4.9.1.0 software. Bar equals 50 μm.

Synchrotron radiation X-Ray fluorescence (SRXRF) is a powerful technique enabling estimates of the average cytoplasmic content and generation of topographical maps (2-D scans) of the spatial distribution of most biologically important elements in individual cultured neurons [17, 18, 24, 25]. As outlined in the Methods section, primary neurons were grown on SiN windows for several days *in vitro*, then plunge frozen, stored cryogenically and were maintained frozen and hydrated during X-ray analysis. Shown in Fig 1A–1F are 2-D SRXRF scans from a well preserved region of a SiN window supporting a culture of primary neurons derived from the dentate gyrus (see Methods) region of the hippocampus. Shown in Fig 1K is a light microscopic image of dentate gyrus neurons prior to plunge freezing. Although the neurons photographed here are not the same as those shown in Fig 1A–1F, the image is taken from the same culture maintained on SiN. Frozen hydrated cultures from the CA1 region and cortex were scanned as well and all images used for data analysis are included in S1 Fig.

Below the top row of images are shown quantitative data (Fig 1G–1J) expressed as μg/cm^2^, derived from a line scan drawn across the soma of a single neuron that purposely included the nucleus (see image 1A with red arrow for approximate location of the line scan). Fig 1A shows the 2-D scan obtained for potassium. Potassium is thought to be uniformly distributed throughout the cytoplasm and nucleus and is present in high concentrations such that detectable fluorescence is obtained even from processes of very small caliber. The potassium scan shows that at the moment of plunge freezing, cellular structures including the complete process network were intact and well preserved throughout the X-ray analysis procedure. Fig 1B shows the 2-D scan for sulfur and adjacent to that is the 2-D scan for zinc (Fig 1C). Like potassium, the sulfur scan is useful, as sulfur is generally accepted to be uniformly distributed throughout the neuron as well. Thus, the sulfur scan, like potassium, generally tracks with the neuron mass profile and thus cellular thickness. By comparing the zinc 2-D scan with potassium and sulfur, it is reasonable to conclude that zinc is uniformly distributed throughout the cytoplasm. The region of the neuron with the largest content of zinc is the nucleus, which overlays well with the potassium and sulfur profiles. These data suggest that the broad peak in zinc, potassium and sulfur content observed in the line scan mostly reflects the thickness of the neuron being scanned. Similar conclusions regarding interpreting SRXRF 2-D scans have been arrived at by others [10, 26]. This conclusion is supported by the data shown in Fig 1G. Here the line scans for potassium and zinc are overlaid. Note that the line scan profiles of each element closely overlap; a reasonable conclusion being that the profiles mostly outline neuronal thickness. However, zinc does show examples of small localized enrichment in the nuclear region.

Calcium, unlike zinc, shows peri-nuclear regions of higher fluorescence intensity and subcellular distribution with clearly discernable peaks (Fig 1D & 1H). The subcellular location of higher calcium fluorescence would likely correspond with regions generally ascribed to the mitochondrial network and endoplasmic reticulum, as shown by the overlaid line scans for potassium and calcium (Fig 1H). Since the calcium level in the nucleus was similar to the level found in non-peak areas of the cytosol and it is known that the volume of the nuclear region is greater than the cytosolic region, these data suggest that the average concentration of calcium was less in the nucleus.

Iron was strikingly different when its subcellular distribution is compared with either zinc or calcium ion (see Fig 1E, 1I & 1J). Fig 1E and 1E1 show iron distributions. Iron, while having a uniform fluorescence in the cytoplasm and nucleus, was found in puncta in a peri-nuclear location with much higher fluorescence intensity. Fig 1E shows the 2-D scan scaled to the entire range of iron values, whereas Fig 1E1 is a blow-up of the region defined by the box in Fig 1E, scaled so that the distribution of iron in the soma can be better visualized. Using such scaling, forces iron puncta to be over-saturated in intensity (see white arrows pointing to red puncta). It’s interesting to note that the average iron contents of measured somata were similar when compared with average zinc contents of the same somata. This indicates that much of the total iron content of an individual neuronal soma is contained within puncta. This is difficult to discern from the 2-D scans (compare Fig 1C with 1E), which are scaled to the fluorescence intensity range of each individual image, and is better demonstrated when the line scans are overlaid (see Fig 1J). Iron levels were similar when comparing the nucleus with the cytoplasm (ignoring iron puncta—compare line scans in Fig 1I with 1J). This finding suggests that average iron concentration, like calcium, is less in the nucleus.

**Fig 2:**
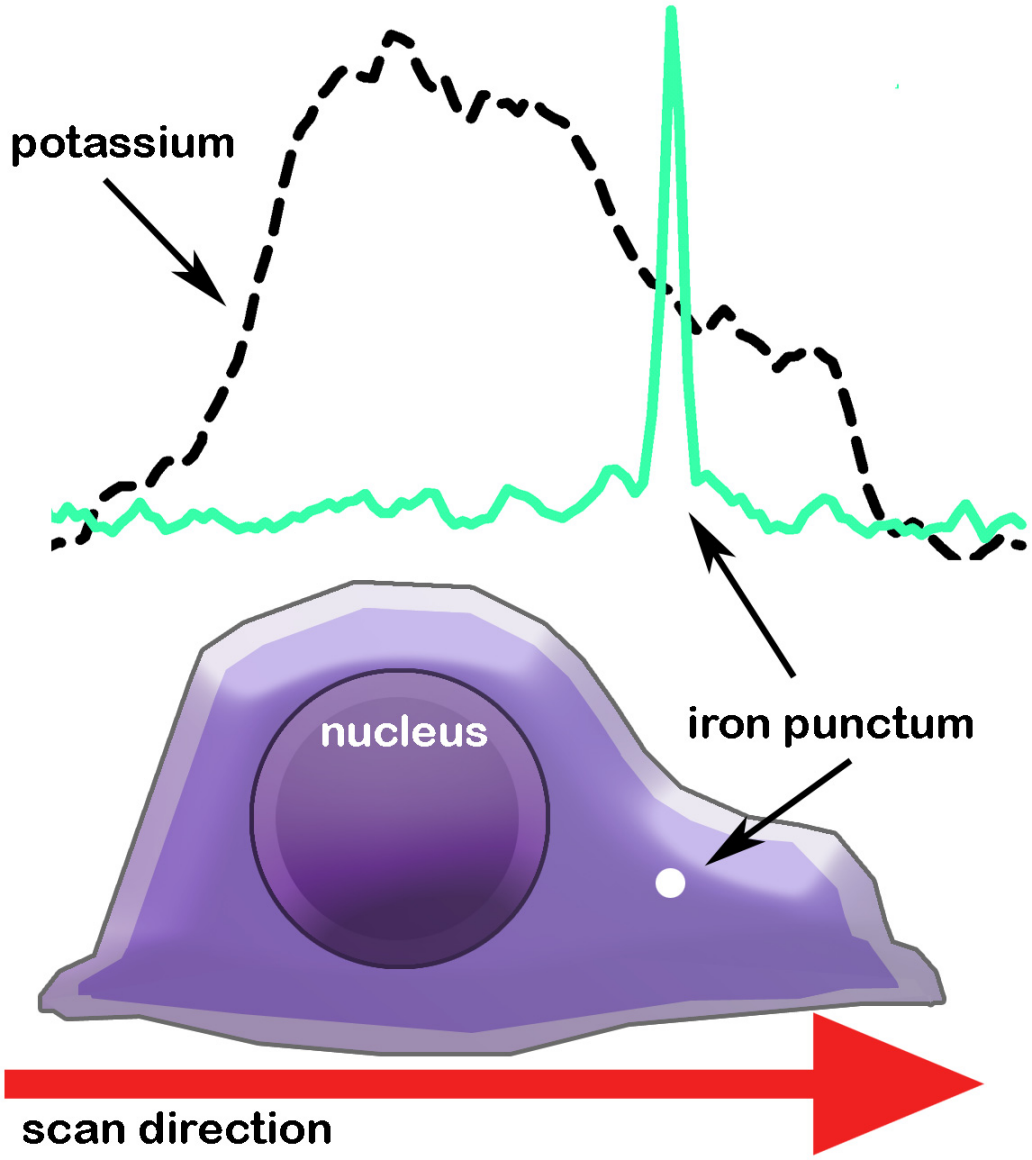
Schematic of an individual neuron soma and how it correlates to line scan fluorescence data. The red arrow depicts the direction of the scan as shown in Fig 1A. The line scan data shown are taken from Fig 1I. A single iron punctum is shown in a peri-nuclear region within the soma.

To better visualize the correlation between line scan data plots and cellular structure a schematic is shown in Fig 2. Here is shown a drawing of a single neuronal soma. Note that the region of greatest thickness is the nucleus. Also shown is the location of an iron punctum. Above the drawing we placed the data from Fig 1I and scaled the x-axis to match the drawing. It can be seen more easily how fluorescence and the calculated average contents of uniformly distributed elements like potassium, sulfur, and zinc would follow the contour (mass) of the soma, in contrast to metals such as iron and calcium that do not.

**Fig 3:**
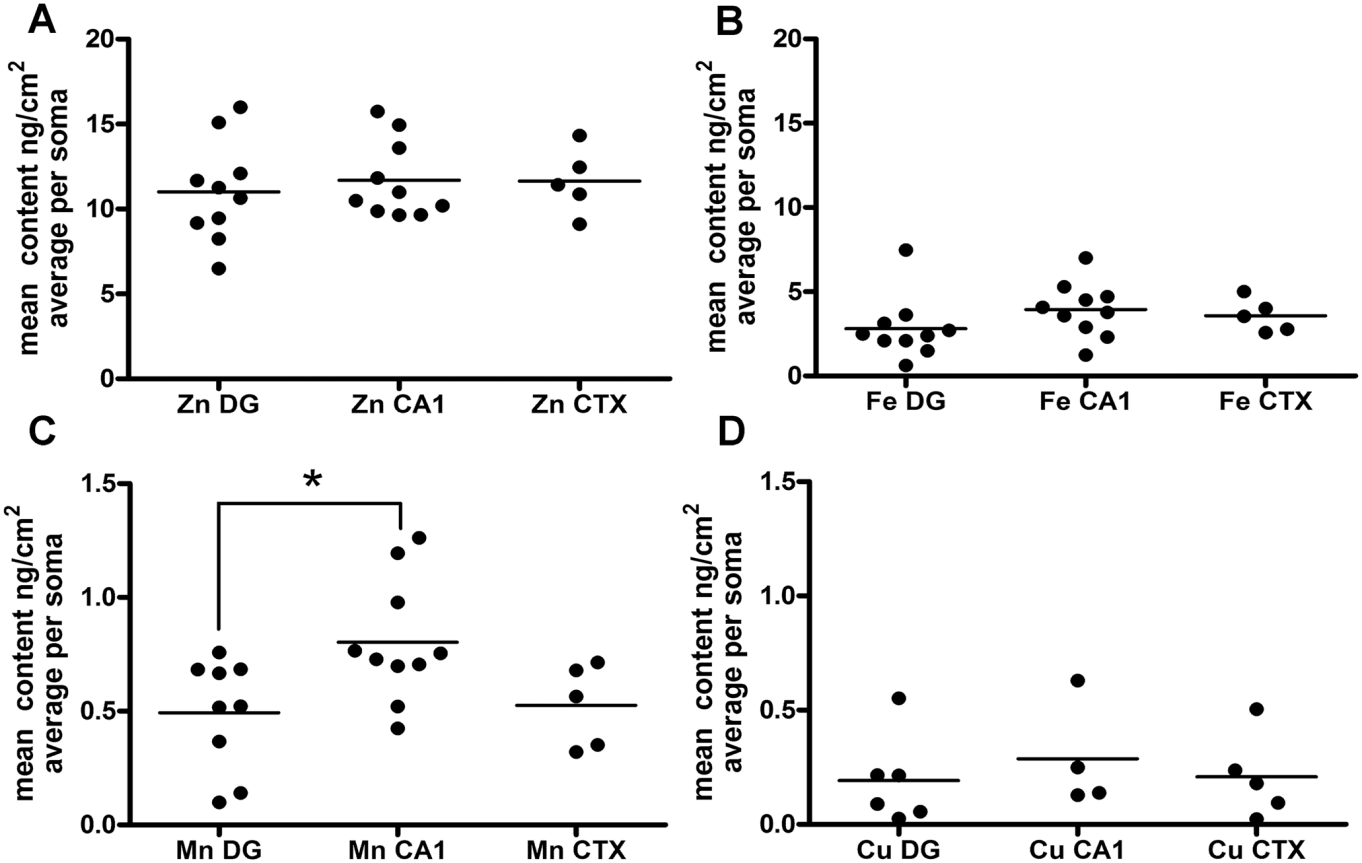
Average metal contents of neuronal soma from hippocampal and cortical primary neurons. Regions of interest (ROI) were manually drawn around neuronal soma observed in 2-D SRXRF scans using MAPS software (see Methods) and metal contents are expressed as the average value for each soma (ng/cm^2^) with sample background subtracted. The data are displayed as scatter plots (horizontal bar represents the mean of all measurements for that experimental condition): (A) zinc, (B) iron, (C) manganese, and (D) copper. Each filled circle represents data obtained from one neuron; an ROI manually drawn around its soma. For each cell type, data were collected from a single SiN window. *—Comparing calculated means for each metal across cell types, only manganese CA1 was found to be significantly greater than manganese dentate gyrus–*p* < 0.05, Kruskal-Wallis test with Dunn's Multiple Comparison Test.

**Fig 4:**
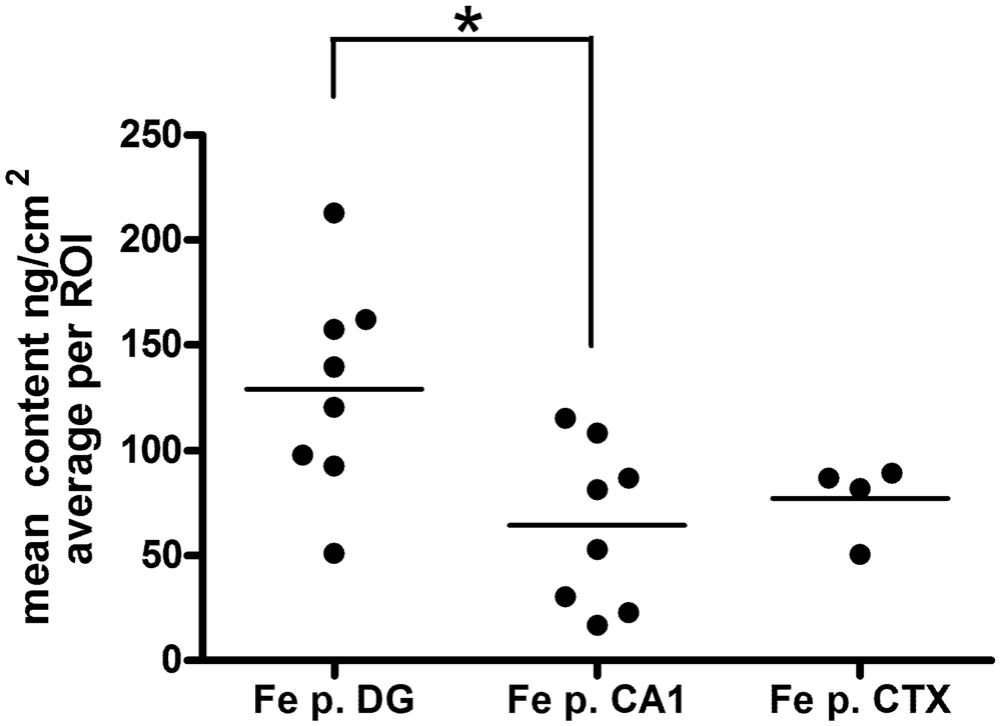
Average iron content in regions of interest (ROI) manually drawn around iron puncta (Fe p., identified in 2-D SRXRF scans; see Fig 1E1 for examples). Metal contents are expressed for each ROI as an average value (ng/cm^2^) with sample background subtracted. The data are displayed as scatter plots (horizontal bar represents the mean of all measurements for that experimental condition), filled circles represent individual iron puncta analyzed for each cell type. Data for iron puncta were collected from 5 DG cells, 7 CA1 cells, and 4 cortical cells. For each cell type, data were collected from a single SiN window. *—Comparing calculated means for each cell type, Kruskal-Wallis test with Dunn's Multiple Comparison Test, *p* < 0.05 for iron puncta (Fe p.) dentate gyrus versus CA1 neurons.

Finally, a 2-D scan for manganese is shown in Fig 1F. Manganese is one of several trace metals that include chromium, cobalt, copper, and nickel, which can be detected in primary cultured neurons by either inductively coupled plasma mass spectrometry or SRXRF [17]. As is typical of all the trace metals just listed, Fig 1F shows that manganese levels are just barely detectable above background fluorescence by single cell SRXRF analysis. Thus, there is little topographical information that can be gleaned from 2-D scans of trace metals, still cellular content in the soma was measured and compared with the abundant metals and other trace metals in the same defined region of interest (see Figs 3 and 4 below). Data for other trace metal levels detectable by SRXRF were collected but levels were close to sample background.

### Analysis of the Average Metal Contents of Cultured Primary Neurons Derived from the Hippocampus and Cortex

Estimates of average metal content (ng/cm^2^) within a single neuron soma or subcellular region were obtained by manually outlining a region of interest (ROI) on a 2-D scan using the MAPS software (see Methods for more detail). Expressing average metal contents as ng/cm^2^ is the direct read-out of surface scans performed in the x-y plane. An estimate of concentration can be made using an average value for the volume of the somata of cultured neurons. It is often reported that the average volume of a cultured neuron soma is about 1,000 to 2,000 μm^3^ (somewhat smaller than cells in intact tissues). Converting the average total zinc content of neuron somata determined in the present study to number of zinc atoms per soma, consistently yields a number between 100 and 200 million atoms. This would equate to an average zinc concentration of about 100 to 200 μM for a neuron soma with a volume of 1,000 μm^3^, consistent with previously published reports [27, 28]. The ROIs outlined manually included either a single neuron soma or iron puncta (iron puncta were described above in the subcellular analysis of metal distributions). Data were collected from primary neurons derived from the dentate gyrus (DG) and CA1 regions of the hippocampus and the cerebral cortex (CTX). Fig 3 shows that no significant differences were found when comparing the zinc, iron, or copper contents in the soma of primary cultured neurons derived from these three brain regions. However, when comparing manganese content across these three brain regions, the observed average manganese levels in neuronal soma derived from CA1 were significantly greater than DG. Using the estimate described above for average soma volume, the average cellular concentration of manganese would be in the range of 1 to 10 μM. Although the average difference between CA1 and CTX manganese concentration did not reach significance the trend was for a lower manganese level in cortical neurons as well, when compared with CA1.

### Analysis of the Average Iron Content of Iron Containing Puncta

Iron puncta identified in iron 2-D scans (see Fig 1E and 1E1) were outlined as ROIs allowing an estimate of their average iron content. The size of iron puncta averaged between 1.5 to 2.0 μm^2^ across all cell types. As noted above, several iron puncta were observed in most neurons. Iron puncta were restricted to a perinuclear location, in contrast, processes were devoid of puncta. We determined that iron levels in iron puncta observed in dentate gyrus neurons were significantly greater than the iron levels in iron puncta found in either CA1 or cortical neurons (see Fig 4). However, the average content of iron determined for neuronal soma was not significantly different when comparing the three cell types (see Fig 3).

## Discussion

Using SRXRF, we generated 2-D metal distribution maps of primary cultured neurons derived from two regions of embryonic rat hippocampus (dentate gyrus and CA1) and cortex and estimated their average metal contents. Our goal was to compare these data to metal distributions already reported for adult tissues to gain new insights into the factors controlling individual metal contents and tissue distributions. Our findings showed that cultured primary neurons from all three regions studied had similar total zinc contents (since each neuron had similar soma volumes) and showed a uniform subcellular distribution of zinc. This finding is consistent with the idea that the amount of free Zn^2+^ accumulated in organelles (like that seen in the cytosol [27, 29]) is negligible when compared with the amount coordinated via thiol and other groups and bound to proteins [19, 29] giving zinc a fairly uniform average concentration throughout the cytoplasm and nucleus of individual somata.

Of particular interest, is the distribution of zinc in the hippocampal formation. Striking differences in zinc levels can be observed in these regions in both human and rodent brains [7, 30]. This unique tissue distribution of zinc has been observed in tissue sections using histochemical methods (Timm’s stain) [31], fluorescent probes [32], and analytical methods such as laser ablation inductively coupled plasma mass spectrometry (LA-ICP-MS) [33–35], synchrotron radiation X-ray fluorescence (SRXRF) [8–10, 15, 36], particle induced X-ray emission (PIXE) [37, 38], and inductively coupled plasma mass spectrometry (ICP-MS) [39]. It is essential to understand that the various methods listed above can and most often do report on levels of functionally distinct pools of zinc within the tissue being assayed. The analytical methods are useful because they simultaneously report levels of total zinc (which includes both bound and free zinc) with total levels for other metals that are present in the same tissue or cellular location. On the other hand, histochemical methods and fluorescent probes report mostly on free or labile zinc levels, although much of the free zinc is understood to be in equilibrium with an extensive buffering system within the neuronal soma [29]. When comparing the data obtained by the various analytical methods listed above, they are in good agreement regarding the relative levels of physiologically important metals such as zinc, iron, calcium, copper, and manganese in the hippocampal formation. Iron, zinc, and calcium are the highest and much lower estimates are obtained for trace metals such as copper and manganese.

The characteristic pattern of zinc seen in the rodent and human hippocampus and cortex with Timm’s stain and fluorescent probes results primarily from the interaction of these molecules with a functionally specialized pool of labile and releasable zinc in synaptic vesicles of glutamatergic nerve terminals. This is confirmed in ZnT-3 zinc transporter knockout animals where this characteristic pattern is nearly abolished. The loss of this specific pool of neuronal zinc amounts to about 20% of the total zinc in this area of the brain [40–42]. The other 80% of brain zinc is located in nuclear and cytoplasmic pools and is highly buffered and almost entirely bound to proteins and other ligands. The average free cytosolic zinc concentration is estimated to be in the picomolar range in spite of the total average concentration of 200–250 μM [17, 27, 28]. The typical cell permeable fluorescent probe has an affinity for free zinc lower than picomolar, so that in resting cells, free cytosolic zinc is nearly undetectable using fluorescent probes. Thus, synaptic vesicle labile zinc concentration must be higher than the free cytosolic concentration such that it can be detected by Timm’s stain and certain fluorescent probes. Quantification of the total zinc in hippocampal sections by SRXRF indicates that the average concentration of total zinc in zinc rich areas of the hippocampus is significantly greater than the average zinc concentration in the rest of the hippocampus [8–10, 15, 36].

Do glial cells contribute to the characteristic distribution of zinc in the hippocampus observed by Timm’s stain and various analytical methods? Glia can accumulate zinc, express ZnT-3, and are involved in brain zinc homeostasis [43]. Glia would be predicted to have average cytoplasmic zinc concentrations similar to neurons and other eukaryotic cells and zinc should be almost entirely protein bound [44]. Although this zinc is not easily detectable with fluorescent probes, it should be detectable with SRXRF in tissue sections. Immunostaining with antibodies to a glial specific protein (glial fibrillary acidic protein) demonstrate that in hippocampal tissue sections the pattern of glial cell distributions can be clearly distinguished from the synaptic organization and neuronal connectivity patterns of the hippocampus, as well as the pattern of zinc distribution. [10]. Thus, the characteristic distribution of zinc in hippocampal sections detectable by SRXRF and Timm’s stain pattern must emanate primarily from neurons not glial cells.

Several analytical methods are available with the necessary sensitivity and resolution limits allowing one to measure even trace metal levels and subcellular distributions (e.g., copper and manganese) in single neurons. Each of these methods have their own pro and cons that primarily revolve around either differences in sensitivity and resolution limits or sample preparation issues, which must be considered when comparing the results of each approach. Several review articles have recently discussed the use of these analytical methods for metal detection in single cells and have compiled data from each method for comparison purposes [3, 4, 6, 10, 45]. SRXRF’s sensitivity limit is exceeded only by LA-ICP-MS and has a resolution limit that allows the detection of subcellular structure. The resolution limit of LA-ICP-MS allows for the analysis of single cells, but is best used for mapping metal distributions in tissue sections. PIXE has a resolution limit similar to SRXRF, but is tenfold less sensitive. Direct comparison of different analytical methods is difficult because of differences in sample preparation and because different units of measurement are used. To the extent that such comparisons can be made, in general, the various analytical methods yield similar results when comparing relative levels of the more abundant metals (e.g., iron, zinc, and calcium) with trace metals (e.g., copper and manganese). The data reported here for average metal contents in neuronal somata derived from CA1, DG, and cortex are in agreement with previously reported data (except where noted below).

When comparing metal levels in primary cultured neurons with adult tissue sections, the culture conditions should be matched as closely as possible to *in vivo* conditions. The ionic composition, pH, osmolality, etc., of body fluids including cerebrospinal fluid are well known and are matched closely by commercially available neurobasal media. The cultured neurons in this study were maintained with serum free supplement B27. The concentrations of factors added to B27 is proprietary and optimized for neuron survival in culture. This mix of supplements does not match the rich environment of neurotropic factors present in the brain during synapse formation In addition, both free and total zinc in the culture media should be considered, the most important being free zinc, since this pool would be the primary source of zinc that differentiating and developing neurons would utilize. It has been determined that free zinc levels in neurobasal/B27 media are approximately 30 nM [46] and free zinc in extracellular fluid of the brain is 5–25 nM [47]. Thus, free zinc concentrations in neurobasal/B27 are very similar to *in vivo* free concentrations. We failed to see significant differences in average zinc content when neurons derived from the CTX, DG and CA1 region of the embryonic brain were cultured separately in neurobasal/B27. Our results suggest that extrinsic factors present in the developing brain are probably critical for initiating and maintaining the accumulation of labile zinc in the synaptic vesicles of glutamatergic nerve endings, since this environment is not easily replicated in primary culture. This conclusion is consistent with the observation that zinc accumulates in the brain during postnatal development [12], which correlates well with the establishment of glutamatergic synaptic contacts. On the other hand, our data would predict that the average zinc content of individual neurons in the hippocampal formation (exclusive of synaptic vesicles) is similar being determined to a greater extent by cellular intrinsic factors and zinc uptake, both being common to neurons in primary culture and mature hippocampus.

In many studies of primary cultured neurons (and other cell types) where zinc-responsive fluorophores (e.g., TSQ, zinquin, ZinPyr-1) are used to visualize intracellular Zn^2+^ using microfluorometry, highly fluorescent presumably zinc-enriched puncta have been observed [48, 49, 50]. Such structures have been termed zincosomes (for review see [51]), but should not be confused with zinc containing synaptic vesicles [7]. We failed to detect zinc puncta in the soma or processes of cultured neurons using SRXRF analysis. When observed with epifluorescence microscopy, cytoplasmic zinquin fluorescent puncta in cultured primary cortical neurons were circular with an average width of 500 nm [49], but their actual size could be significantly smaller. The digital spatial resolution limit of SRXRF at the 2-ID-D beamline at APS is the result of Fresnel zone plates that focus X-rays from a high brightness undulator source to a 200–400 nm spot on the sample and the scan step size (250 nm in all experiments). Thus, we expected to observe zincosomes by SRXRF if their actual size was indeed 500 nm or greater. Although we could find no evidence for the existence of cytoplasmic zincosomes by SRXRF, this observation does not preclude the existence of zincosomes smaller than the digital spatial resolution limit of SRXRF.

A likely explanation for the observance of zinc puncta when using zinc-responsive fluorophores, but not when using SRXRF, is that zincosomes may preferentially accumulate zinc-responsive fluorophores and increased fluorescence intensity mostly reflects fluorophore accumulation. For example, a strong ZinPyr-1 fluorescence can be found in lysosomes even if all chelatable Zn^2+^ is sequestered by TPEN [52]. Thus, although the structures exist and contain zinc, the average zinc concentration in these structures is similar to the average concentration in the cytosol, nucleus, and other organelles and thus undetectable by SRXRF.

Finally, we should discuss the possibility that sample processing disturbed cellular ultra-structure enough to negatively influence the detectability of zincosomes. We have shown that radiation damage during X-Ray scanning is minimal [24], but a change in cellular ultra-structure during plunge-freezing and storage of the cells under liquid nitrogen cannot be absolutely excluded either. Plunge freezing and keeping the neurons cryogenically preserved in a fully hydrated state provides the optimal conditions to minimize disruptions to cellular structure and this approach has been used by others to preserve cellular ultra-structure [6, 53].

Iron puncta or siderosomes characterized by ultrastructural analyses of tissues subjected to iron overload [54] are circular and average 1 μm in diameter [55]. We were able to detect iron puncta of similar size by SRXRF, which is safely above the digital spatial resolution limit of SRXRF. Iron puncta have been observed previously using SRXRF in rodent cultured primary cortical and midbrain dopaminergic neurons and in hippocampal tissue sections [10, 17, 18, 25, 56]. Typically, scanned neurons exhibit one to several iron puncta. But, morphologically, there is no evidence that primary cultured neurons are suffering from iron overload [17]. We have suggested that primary neurons maintained in neurobasal and B27 supplement (see Methods) take up iron from the media accumulating iron in one to several siderosomes of similar size [17]. Iron taken up by neurons in the brain is thought to be mostly transferrin bound [57] and B27 supplement includes transferrin [58]. The concentration of iron in neurobasal media (Invitrogen, ThermoFisher) is 250 nM, but with 2% B27 supplement added, the total iron concentration in the culture media increases 10 fold above that value [27].

The greater capacity of dentate gyrus neurons to store iron in siderosomes (with no change in total cellular iron content and no apparent deleterious effects) could be interpreted as a protective mechanism against redox damage that could result if cytosolic levels of iron were allowed to rise producing damaging reactive oxygen species within the cell [59–61]. It is well established that much cytosolic iron is stored within ferritin cages protecting the cell from potentially harmful redox reactions [62]. Siderosomes may be a second mechanism by which neurons can store cytosolic iron in a non-reactive form. Both storage mechanisms may have implications for human neurodegenerative diseases as experimental evidence suggests that iron levels increase in the hippocampus of Alzheimer’s disease patients [63–66] and with normal aging [67]. Iron chelation has been shown to reverse age related cognitive deficits in rats [68].

The hippocampus contains measureable amounts of manganese, but a specialized role of manganese specific for hippocampal neurons has not been identified [69]. Many cellular mechanisms exist that regulate the influx and efflux of manganese in neurons, which are designed to maintain intracellular levels within narrow limits [70]. Manganese is thought to function primarily as a cofactor for enzymes critical for normal physiology and development. Any function ascribed to manganese should take into consideration that levels are much lower than abundant metals like iron, zinc, or calcium and more similar to levels of other trace metals like copper or nickel [17, 39].

In hippocampal and dopaminergic neurons exposed to manganese in culture, manganese appears to accumulate in a peri-nuclear location likely to include the Golgi apparatus [52, 71, 72]. Because of low levels of manganese detected in the present study, a convincing peri-nuclear spatial distribution in resting primary neurons was not obtained. We observed that CA1 neurons in primary culture exhibited a greater mean average manganese content than DG or CTX neurons. Previous studies using mature hippocampal slices found the CA3 region had the highest manganese content. However, we did not study primary neurons derived from the CA3 region. Our studies will require additional replicates and a larger sample size before any conclusions can be made regarding their significance and implications for regional differences in manganese and hippocampal function.

## Conclusions

In this study, we used SRXRF to study average contents of the soma and intracellular spatial distributions of several metals in primary cultured neurons from the hippocampal formation and cortex. In the mature hippocampus and cortex, distinctive distributions of metals such as zinc, iron, and manganese have been observed. Particularly for zinc, these distributions are thought to be related to the synaptic function of these brain regions. We sought to determine if the different metal contents observed in these brain regions were intrinsic to the neurons comprising each region by determining the average metal contents of primary neurons cultured from embryonic tissues. Although our conclusions are limited by a small sample size, we found few differences in the mean average metal contents of primary neurons cultured from the dentate gyrus or CA1 regions of the hippocampus and cortex. We did find that manganese levels were somewhat higher in the CA1. On the other hand, we found characteristic and distinct intracellular spatial distributions when analyzing individual neurons from these regions. Zinc was uniformly distributed. Calcium showed a peri-nuclear distribution that could be best explained by accumulation in endoplasmic reticulum and mitochondria. Iron showed distinct high concentration puncta in peri-nuclear locations; in the rest of the cytoplasm, iron concentration was much lower and distributed uniformly. Iron puncta contained the highest levels of iron in DG when compared with CTX or CA1. Our results are consistent with the belief that metal distributions in mature brain structures are most likely influenced by both the intrinsic properties of the neurons and extrinsic factors, related to synapse formation postnatally and maintenance of synaptic organization and contacts in the mature tissue.

## Supporting Information

S1 Fig 2-D scans for all cells used in the metal analyses reported in the manuscript. Each image series is labeled CA1, DG, or cortical (CTX) and each scan is labeled with the metal it represents. Images are not background corrected.
(PDF)
