# Supplementary material for: Visualizing Metal Content and Intracellular Distribution in Primary Hippocampal Neurons with Synchrotron X-Ray Fluorescence
Source: PLoS One. 2016 Jul 19;11(7):e0159582. doi: 10.1371/journal.pone.0159582 (PMC4951041; doi:10.1371/journal.pone.0159582)

S1 Figure: Supporting Information for: Visualizing metal content and intracellular distribution in primary hippocampal neurons with synchrotron X-ray fluorescence.

2-D scans for all cells used in the metal analyses reported in the manuscript. Each image series is labeled CA1, DG, or cortical (CTX) and each scan is labeled with the metal it represents. Images are not background corrected.

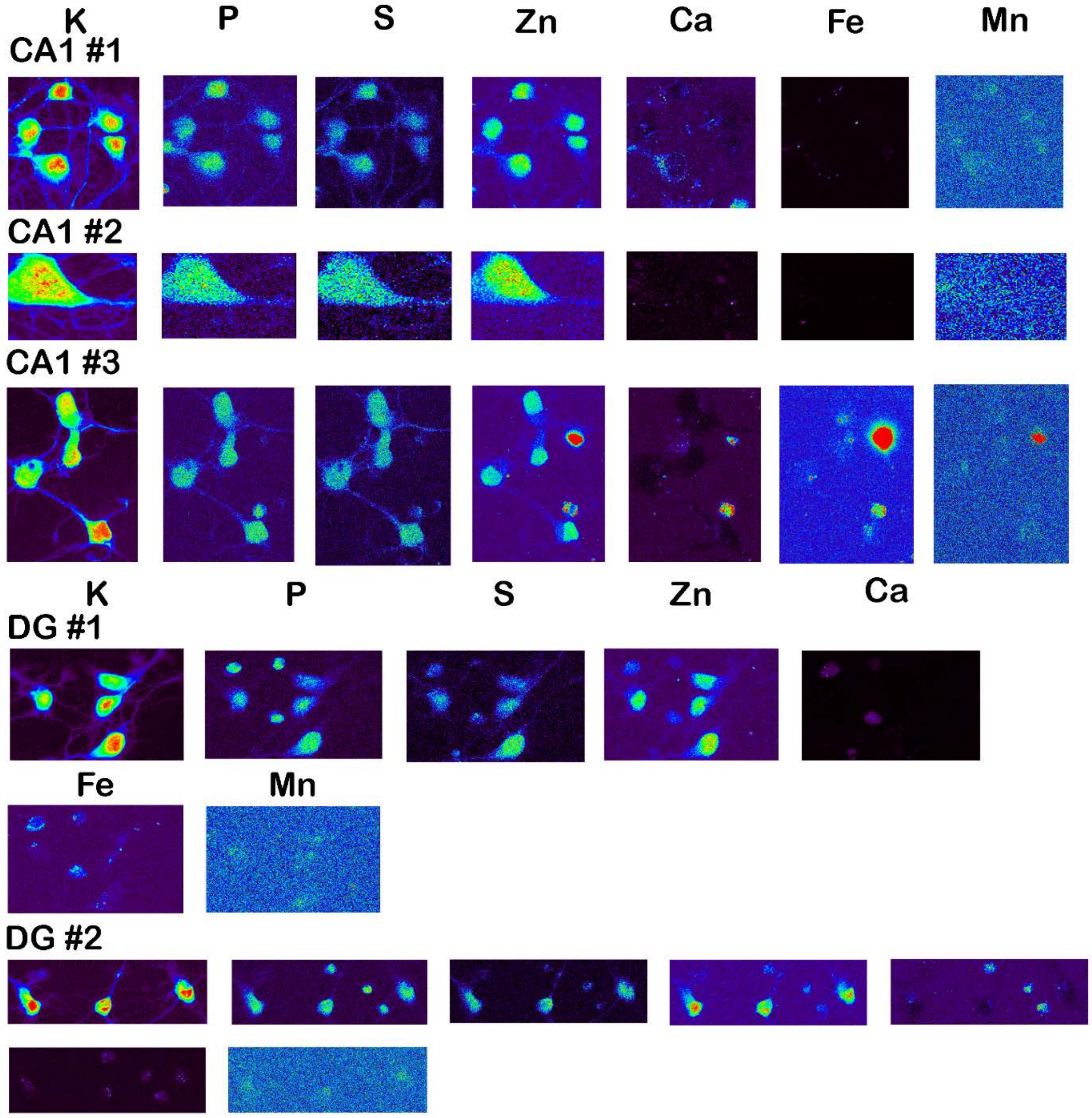

2-D scans continued:

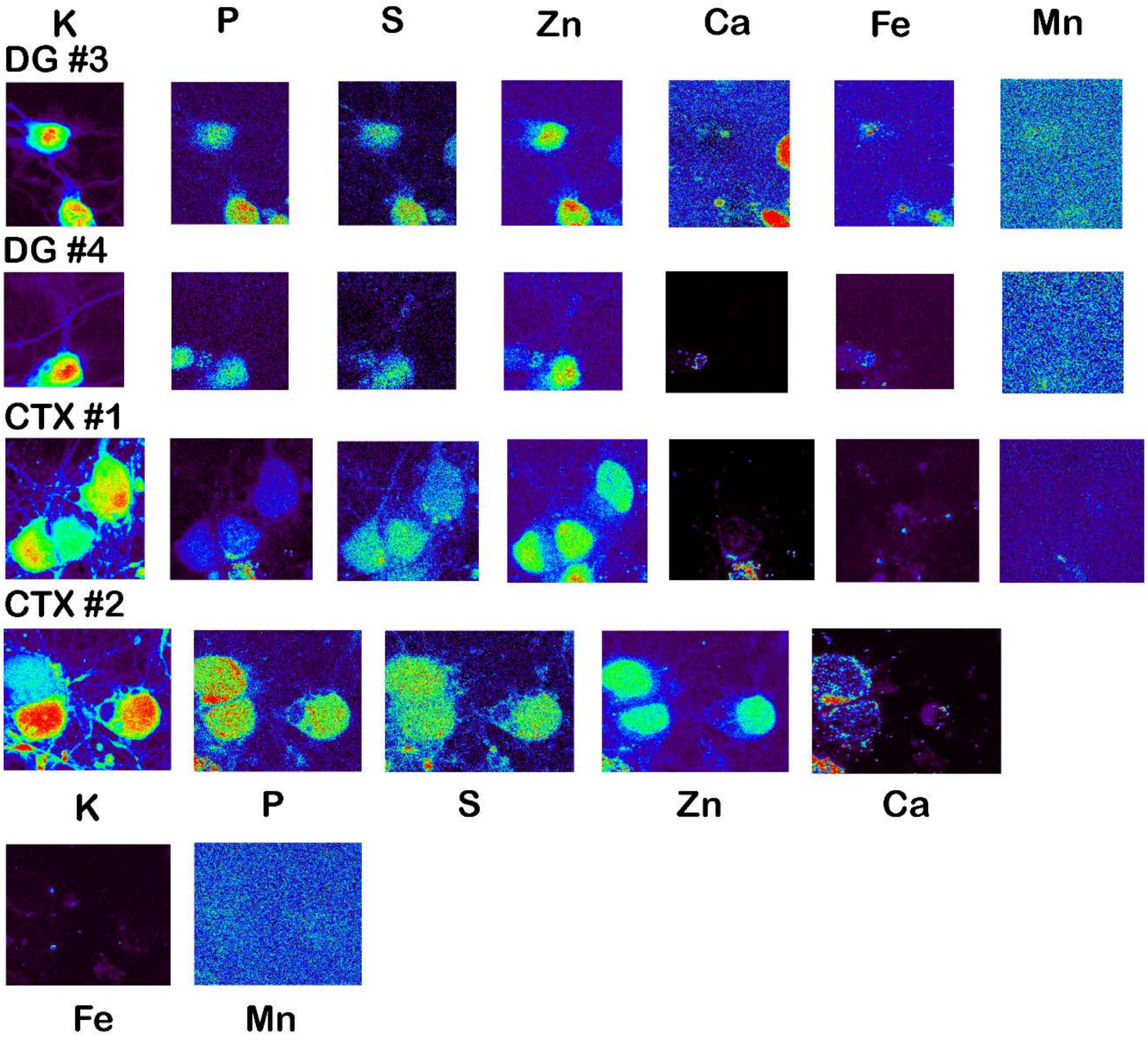

Supplement: S1 Fig — Each image series is labeled CA1, DG, or cortical (CTX) and each scan is labeled with the metal it represents. Images are not background corrected. (PDF) [file pone.0159582.s001.pdf]
